# 15d-PGJ2 Reduced Microglia Activation and Alleviated Neurological Deficit of Ischemic Reperfusion in Diabetic Rat Model

**DOI:** 10.1155/2015/864509

**Published:** 2015-12-30

**Authors:** Lihong Huang, Gang Li, Xiaofang Feng, Luojun Wang

**Affiliations:** ^1^Department of Neurology, Central Hospital of Zhabei District, Shanghai 200070, China; ^2^Department of Neurology, East Hospital, Medicine School of Tongji University, Shanghai 200120, China

## Abstract

To investigate the effect of PPAR*γ* agonist 15d-PGJ2 treatment on the microglia activation and neurological deficit of ischemia reperfusion in diabetic rat model, adult Sprague-Dawley rats were sacrificed for the research. The rats were randomly categorized into four groups: (1) sham-operated group; (2) standard ischemia group; (3) diabetic ischemia group; (4) diabetic ischemia group with diabetes and treated with 15d-PGJ2. Compared to the sham-operated group, all the ischemic groups have significantly severer neurological deficits, more TNF-*α* and IL-1 expression, increased labeling of apoptotic cells, increased CD68 positive staining of brain lesion, and increased volume of infarct and cerebral edema in both 24 hours and 7 days after reperfusion. Interestingly, reduced neurological deficits, decreased TNF-*α* and IL-1 expression, less apoptotic cells and CD68 positive staining, and alleviated infarct and cerebral edema volume were observed when 15d-PGJ2 was intraperitoneally injected after reperfusion in diabetic ischemia group, suggesting its neuroprotective role in regulating microglia activation, which may have a therapeutic application in the future.

## 1. Introduction

The activation of microglia has been observed in the ischemia patients decades ago. And there is a neuroprotective effect in nondiabetic stroke patients by inhibiting its activation [1–5]. The activation of microglia is also involved in the inflammation [6] and phagocytosis of dying neurons in the diabetes-caused stroke [7–9]. The peroxisome proliferator activated receptor *γ* (PPAR*γ*) and its agonist are very essential for regulating the response of ischemia and inflammation after stroke, especially in regulating the cell proliferation, differentiation, and apoptosis [10, 11]. The activation of microglia is significantly inhibited by upregulation of PPAR*γ* expression. And the proliferation of microglia is also inhibited upon PPAR*γ* activation by regulating the cell cycle related genes. Victor et al. have shown that the mRNA is highly increased 24 hours after focal ischemia brain damage in nondiabetic rats. And the damaged area of ischemic stroke was significantly reduced after PPAR*γ* agonist intervention while it was significantly increased by the PPAR*γ* antagonist modulation [12]. 15-Deoxy-Δ12,14-Prostaglandin J2 (15d-PGJ2) is a natural ligand of PPAR*γ*. Xu and Pan have shown that the activation of microglia can be inhibited by 15d-PGJ2 in the experimental autoimmune encephalomyelitis (EAE) mouse model [13]. They also found that the release of proinflammatory cytokines was slowed and the neural function was improved. This finding is also validated in the osmotic demyelination mouse model by Takefuji et al. when the activation of microglia was inhibited by lovastatin [14]. In our research, the intraluminal middle cerebral artery occlusion (MCAO) was induced in a diabetes mouse model. And the activation of microglia was investigated after intraperitoneal 15d-PGJ2 injection. Meanwhile, the neuron apoptosis, area of brain lesion, and neurological deficit were evaluated before and after stroke induction.

## 2. Materials and Methods

### 2.1. Materials

The 2% 2,3,5-triphenyltetrazolium chloride (TTC) staining solution was obtained from Beijing Leagene Biotech. Co., Ltd. (Cat. number DK0005, Beijing, China). Streptozotocin (STZ, Cat. number S0130) and 15d-PGJ2 (Cat. number 87893-55-8) were from Sigma (St. Louis, MO, United States) and Santa Cruz Biotechnology (Shanghai) Co., Ltd., respectively. CD68 antibody (KP1) was produced by Abcam (Cat. number ab995) and transferase-mediated deoxyuridine triphosphate-biotin nick end labeling (TUNEL) kit was produced by Roche (Cat. number 11684817910); ELISA kits for detecting TNF-*α* (Cat. number 1R350) and IL-1*β* (Cat. number 1R040) were from RapidBio.

### 2.2. Methods

#### 2.2.1. Animal Models and Experimental Groups

All the rats were provided by Shanghai Bikai Lab Animal Research Center. One hundred and twenty adult Sprague-Dawley rats (12 weeks old and weight 200–250 g) were sacrificed for the research. The rats were randomly categorized into four groups: (1) sham-operated group; (2) standard ischemia group; (3) diabetic ischemia group; (4) diabetic ischemia group and treated with 15d-PGJ2. All procedures in this study were in accordance with the local guidelines for the care and use of experimental animals.

The rats of sham-operated group were operated on without injection of STZ and ischemia induction. The rats of standard ischemia group were modeled according to a modified Zea-Longa protocol. Briefly, under general anesthesia (10% chloral hydrate; 0.3 mL/kg), the right common carotid artery (CCA), external carotid artery (ECA), and internal carotid artery (ICA) were exposed. Subsequently, the distal end of right ECA was ligated while the branch vessels were sealed by heat. An 18–20 mm long monofilament nylon suture thread with a diameter of 0.24–0.26 mm was inserted into the right internal carotid artery via the external carotid artery until a slight resistance was encountered while the CCA and ECA were blocked by clips. The suture was left in place for 120 min and removed to facilitate the reperfusion. The sham-operated rats were modeled with the same procedure as above but not inserting the suture thread.

The diabetic rats (3 months old and 200–250 g weight) were modeled by intraperitoneally injecting a low dose of streptozotocin (STZ) (60 mg/kg) which was previously dissolved in 0.05 mol/L sodium citrate (PH 4.6) to reach a concentration of 1%. The animals had free access to food and water after the STZ injection. After 48 h after the STZ injection and an overnight fast, the presence of diabetes was verified by blood glucose concentrations above 16.65 mmol/L, as determined by using a blood glucose monitor in samples obtained from the tail veins, and the concentration of urine glucose is greater than 110 mM. And the rats of diabetic ischemia group were modeled in diabetic rats with modified Zea-Longa protocol described above.

As for the 15d-PGJ2 treatment, the diabetic rats were intraperitoneally injected with 15d-PGJ2 200 *μ*g/kg/d for 21 days before ischemia induction. 400 *μ*g/kg 15d-PGJ2 was injected 3 hours after reperfusion. And 15d-PGJ2 200 *μ*g/kg/d was injected in the following 6 days. The same amount of 0.9% sodium chloride solution was injected intraperitoneally to the control animals with the same procedure. And the rats were sacrificed 24 hours later or 7 days after the reperfusion as indicated in the research.

#### 2.2.2. Neurological Deficits Evaluation

The neurologic deficits of the mice were evaluated according to the criteria of Longa 5 scores. The higher the score, the severer the neurological deficit (see Table 1).

#### 2.2.3. Tissue and Slice Preparation

The brain tissue was isolated 24 hours or 7 days after reperfusion. For detecting the CD68 positive cells and analyzing the TNF-*α* and IL-1*β*, the tissue was dissected from both proximal and distal regions of the ischemia brain on ice. The proximal ischemia region was defined as 7–11 mm posterior to the tip of olfactory bulb and one-third from the middle to lateral of the hemisphere, while the distal ischemia region is the same as the proximal ischemia region in the contralateral hemisphere.

The brain tissue 3 mm away from both posterior and anterior of the optic chiasm was cut and fixed by 10% formalin before being embedded by paraffin. And the paraffin-embedded tissue was cut coronary and consecutively at 3 *μ*m followed by TUNEL assay. And the brain tissue for TTC staining was cut at 2 mm consecutively for 5 slices by cryostat after being frozen at −20°C for 15 min.

#### 2.2.4. Immunohistochemistry for Detecting Microglia Activation

The microglia activation was evaluated by analyzing the CD68 positive area. The paraffin-embedded sections were stained with primary antibody CD68 and visualized by staining with DAB. Four images from both proximal and distal ischemia regions were selected for image analysis by IMS image analysis software (JRDUN Biotechnology Co. Ltd.). Briefly, the CD68 positive stained region was selected by the software automatically. And then the nonspecific area was erased manually. The area (*μ*m^2^) of the CD68 positive region was calculated by the software.

#### 2.2.5. ELISA Assay for Analyzing Proinflammatory Factors

The ischemia tissue of the brain was homogenized on ice after being quickly frozen in liquid nitrogen and the supernatant was collected after centrifuging at 2000–3000 rpm for 20 min. The amount of TNF-*α* and IL-1*β* was measured by ELISA.

#### 2.2.6. TUNEL Assay

The TUNEL assay was performed according to the protocol recommended by manufacturer to analyze the apoptotic cells. The DNA breaks were detected by labeling the free 3′-OH termini with biotin-dUTP and visualized by DAB. PBS instead of primary antibody was included in the assay as a negative control. Apoptosis was evaluated on the 3 *μ*m paraffin-embedded brain slice. The TUNEL-positive cells were counted in five randomly selected areas per slice and the percentage of positively stained cells was calculated against total nuclei in the selected area by IMS image analysis software (JRDUN Biotechnology Co. Ltd.).

#### 2.2.7. Quantification of Infarct Volume and Cerebral Edema Volume of Focal Cerebral Ischemia

The brain slices were stained in 2% TTC solution for 30 min at 37°C in a dark chamber before fixing with 4% polyformaldehyde for another 24 hours. The images of the brain slices were analyzed by AUTOCAD2000 (Autodesk). And the lesion volume (*V*) was calculated according to the formula: *V* =* t* × (*A*1 +* A*2 +* A*3 +* A*4 +* A*5), in which *t* means thickness of the slice (2 mm) and* A*1–*A*5 mean the area of five representative consecutive slices of ischemia. The ipsilateral and contralateral hemisphere of the ischemia brain were indicated as *V*1 and *V*2, respectively. And the infarct volume (*V*3) was calculated as *V*2 minus *V*1 (mm^3^) while the cerebral edema volume (*V*4) was equal to *V*1 minus *V*2 (mm^3^).

#### 2.2.8. Statistical Analysis

The statistical analysis was performed by SPSS (Version 13.0, SPSS Inc., Chicago, IL). Data were presented as means ± standard deviation and the difference was analyzed by one-way ANOVA followed by Bonferroni's post hoc test. Wilcoxon test was used for heterogeneity test of variance. The difference was considered as statistically significant when *P* value was less than 0.05.

## 3. Results

### 3.1. Neurological Deficits Evaluation

The neurological deficits of the mice were evaluated by Longa 5 scores. Compared to the sham-operated group, all the ischemic groups have significantly neurological deficits indicated by higher Longa score (*P* < 0.05, Table 2). And the neurological deficits were significantly severer in the diabetic ischemia group than that of standard ischemia group with normal level of blood sugar (*P* < 0.05, Table 2) in both 24 hours and 7 days after reperfusion. Interestingly, the severity of the neurological deficits was reduced when 15d-PGJ2 was intraperitoneally injected after reperfusion in diabetic ischemia group.

### 3.2. Evaluation of Microglia Activation

**Figure 1:**
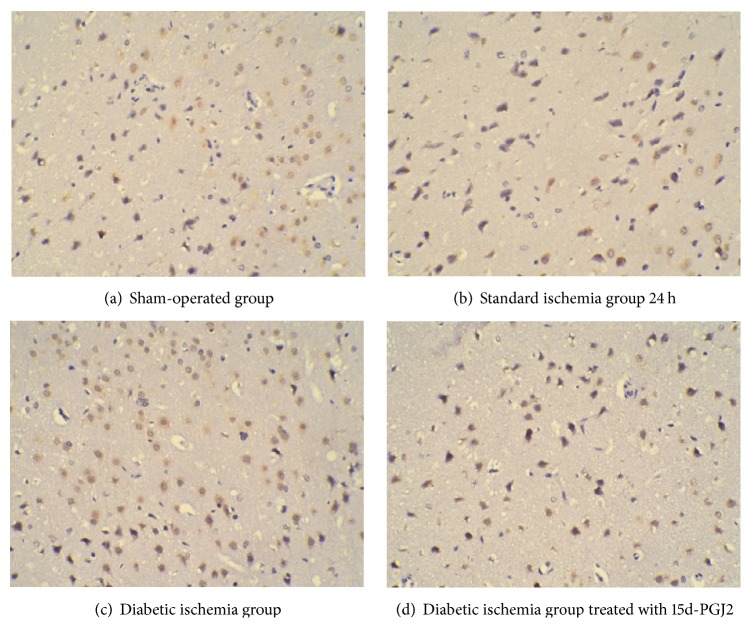
Representative CD68 staining related to Table 3. The paraffin-embedded sections were stained with CD68 antibody and visualized by DAB staining (brown). As indicated, the representative staining of the brain slices from different group was shown in (a), (b), (c), and (d), respectively.

The microglia activation of the mice was investigated by measuring the area of CD68 positive cells (*μ*m^2^). Compared to the sham-operated group, all the ischemic groups have significantly more microglia activation (*P* < 0.05, Table 3). And there is more microglia activation in the diabetic ischemia group than that of standard ischemia group (*P* < 0.05, Table 3) in both 24 hours and 7 days after reperfusion. Consistent with the observation of neurological deficits, the activation of the microglia was reduced when 15d-PGJ2 was intraperitoneally injected after reperfusion in diabetic ischemia group, indicating its neuroprotective role for ischemia. And representative immunohistochemistry slices 24 hours after reperfusion were shown in Figures 1(a), 1(b), 1(c), and 1(d).

### 3.3. Inflammatory Factors Expression

The microglia can activate the proinflammatory factors such as TNF-*α*, IL-1 upon activation. To test whether the activated microglia could trigger inflammatory reaction, we measured TNF-*α*, IL-1 expression level in the ischemia damaged brain tissue by ELISA. Compared to the sham-operated group, all the ischemic groups have significantly more TNF-*α* and IL-1 expression (*P* < 0.05, Tables 4 and 5). And there is more TNF-*α* and IL-1 expression in the diabetes ischemia group than that of standard ischemia group with normal level of blood sugar (*P* < 0.05, Tables 4 and 5) in both 24 hours and seven days after reperfusion. Consistent with the observation in neurological deficits and microglia activation, the proinflammatory factors TNF-*α* and IL-1 expressions were reduced when 15d-PGJ2 was intraperitoneally injected after reperfusion in diabetic ischemia group.

### 3.4. Evaluation of Apoptosis

**Figure 2:**
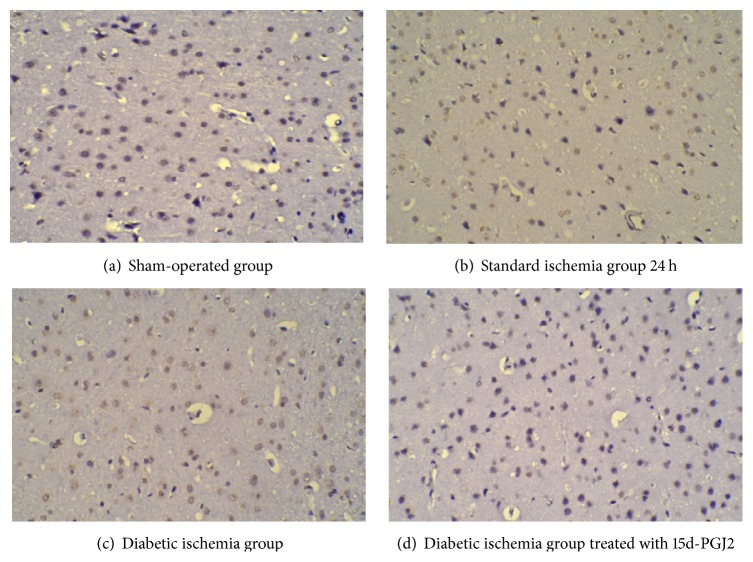
Representative TUNEL assay related to Table 6. The paraffin-embedded sections were stained with biotin-dUTP and visualized by DAB (brown). As indicated, the representative staining of the brain slices from different group was shown in (a), (b), (c), and (d), respectively.

Microglia-produced TNF-*α* causes neural tissue to undergo apoptosis and increases inflammation. To investigate the apoptosis caused by microglia activation in different ischemia models, we performed TUNEL assay. Compared to the sham-operated group, all the ischemic groups have significantly increased labeling of apoptotic cells (*P* < 0.05, Table 6). And there is more apoptotic cells in the diabetic ischemia group than that of standard ischemia group with normal level of blood sugar (*P* < 0.05, Table 6 and Figure 2) in both 24 hours and 7 days after reperfusion. The apoptotic cells decreased when 15d-PGJ2 was intraperitoneally injected after reperfusion in diabetic ischemia group.

### 3.5. Quantification of Infarct Volume and Cerebral Edema Volume of Focal Cerebral Ischemia

To evaluate how severe the brain damage is, the volumes of infarct area and cerebral edema were measured in different ischemia mouse models. And the ischemic brain tissue was stained by 2,3,5-triphenyltetrazolium chloride (TTC) to indicate area of brain lesion. Compared to the sham-operated group, all the ischemic groups have significantly increased volume of infarct and cerebral edema (*P* < 0.05, Table 7). And there is increased volume of infarct and cerebral edema in the diabetic ischemia group than that of standard ischemia group with normal level of blood sugar (*P* < 0.05, Table 7) in both 24 hours and 7 days after reperfusion. Consistent with the observation in neurological deficits and microglia activation, the lesion area decreased when 15d-PGJ2 was intraperitoneally injected after reperfusion in diabetic ischemia group.

## 4. Discussion

The microglia were firstly described by Rio-Hortega as a special type of glia cells in 1919. Microglia are derived from myeloid monocyte and considered as immune cells of the central nervous system (CNS) which are very important for regulating the microenvironment. Microglia accounts for 10% of the total neural cells of CNS and 20% of the glia cells of the brain in adult human. Microglia cells distribute heterogeneously in both grey matter and white matter of the brain, widely enriched in the hippocampus, basal ganglia, and substantia nigra and rare in brainstem and cerebellum. The morphology of the microglia is diverse and usually depends on their location to the vasculature and physiological conditions. Normally, microglia compose a small cellular body with branching processes and less cytoplasm, as well as a poor antigen presenter when they are resting or surveying. The microglia sense and survey the microenvironment by constantly moving their processes and scavenge the apoptotic cells [15]. Not only the morphology and function of microglia but also genes expression profile changes greatly and rapidly when they are activated by external stimuli. The stimuli could be a variety of pathological factors, such as ischemia, infection, or injury. The activated microglia can home to the lesion site by modifying the way of their processes movement [16].

In our research, we compared all the ischemia groups to the sham-operated group and found that ischemia groups have significantly severer neurologic deficits, more TNF-*α* and IL-1 expression, increased labeling of apoptotic cells, increased CD68 positive staining of brain lesion, and increased volume of infarct and cerebral edema (*P* < 0.05) in both 24 hours and 7 days after reperfusion. We think it is because that the neurotoxic substance such as inflammatory factors released by activated microglia after ischemic injury triggered a cascade of response that can make further damage of the brain. The acute inflammatory response breaks the brain-blood-barrier and induces brain edema and neural cell death. The neural cell death is programmed (apoptotic) [17] and determines the infarct volume [18]. Li et al. show that the number of apoptotic neurons increased in the high blood glucose ischemia diabetic reperfusion model [19]. And our research confirmed that apoptosis increased in diabetic ischemia group than standard ischemia group in both 24 hours and 7 days after reperfusion modeling. Therefore, antiapoptosis therapy may be an option for the treatment of ischemia.

The peroxisome proliferator activated receptor *γ* (PPAR*γ*) is involved in the pathological progression in the neural cell apoptosis after ischemia lesion [20]. The PPAR*γ* is expressed mainly in microglia and astrocytes in the brain [21]. The agonist of PPAR*γ* has been extensively investigated for its anti-inflammatory and neuroprotection in brain ischemia, spinal injury and other CNS injuries, experimental autoimmune encephalomyelitis, Parkinson's disease, and other neurodegenerative diseases. The ligands of PPAR*γ* could be from nature or synthetic [22]. 15-Deoxy-Δ12,14-Prostaglandin J2 (15d-PGJ2) is a natural ligand of PPAR*γ* [23]. Its binding to PPAR*γ* can inhibit the immune response in ischemic reperfusion brain thereby mitigating pathological damage [24]. 15d-PGJ2 can also restrain the autophagy of neurons in the ischemia lesion by upregulating Bcl-2 [25]. Recently, reports show that 15d-PGJ2 can prevent myocardiocyte and hepatocyte from apoptosis via regulating the expression of inflammatory factors [26, 27]. The inflammatory response in the CNS is characterized by the proliferation and activation of microglia and astrocytes. The overactivation of microglia after diabetic stroke promotes the secretion of proinflammatory factors. Therefore, we focused on the question whether the apoptosis of neural cells could be decreased by inhibiting the microglia activation and inflammatory response. And we found that 15d-PGJ2 can reduce microglia activation and alleviate neurological deficit in diabetic ischemia reperfusion rat model by blocking the secretion of inflammatory cytokines, preventing the neuron entering apoptosis. And the infarct volume and cerebral edema volume were also reduced upon 15d-PGJ2 suggesting its neuroprotective role in regulating microglia activation.

PPAR*γ* is involved in a variety of physiological and pathological processes, including lipid acid metabolism, cell proliferation and differentiation, inflammatory and immunological response, oxidative stress, and tumorigenesis. The upregulation of PPAR*γ* is not only related to the inflammatory injury of brain ischemia but also related to the vascular inflammation. The upregulated PPAR*γ* can prevent atherosclerosis by improving the endothelial cells function, inhibiting the proliferation and migration of vascular smooth muscle, and stabilizing the plaques [28–31]. And our research shows that 15d-PGJ2 can inhibit immune response and alleviate ischemic injury in the reperfused organs by activating PPAR*γ* which could be a new target for cerebrovascular disease treatment.

## Figures and Tables

**Table 1:** Criteria of Longa 5 scores.

Score	Symptom
0	Normal walk
1	Inability to extend contralateral forelimb
2	Circling toward the contralateral side
3	Fall to the contralateral side
4	Inability to walk and unconsciousness

**Table 2:** Comparison of neurological deficits score.

Group	Neurological deficits scores	
	24 hours after reperfusion	7 days after reperfusion
Sham-operated group	0	0
Standard ischemia group	3.15 ± 1.23^△^	2.76 ± 1.57^△^
Diabetic ischemia group	3.44 ± 0.58^△*∗*^	3.01 ± 0.94^△*∗*^
Diabetic ischemia group and treated with 15d-PGJ2	2.63 ± 0.82^△*∗*▲^	2.12 ± 1.01^△*∗*▲^

Note: △ indicates *P* value is less than 0.05 when compared with sham-operated group; *∗* indicates *P* value is less than 0.05 when compared with standard ischemia group; ▲ indicates *P* value is less than 0.05 when compared with diabetic ischemia group.

**Table 3:** Evaluation of microglia activation.

Group	CD68 positive area (*μ*m^2^)	
	24 hours after reperfusion	7 days after reperfusion
Sham-operated group	203 ± 45.40	196 ± 102.70

Standard ischemia group	662 ± 112.56^△^	574 ± 134.20^△^
Diabetic ischemia group	856 ± 178.09^*∗*△^	743 ± 205.67^*∗*△^
Diabetic ischemia group and treated with 15d-PGJ2	587 ± 155.65^*∗*▲△^	405 ± 98.62^*∗*▲△^

Note: △ indicates *P* value is less than 0.05 when compared with sham-operated group; *∗* indicates *P* value is less than 0.05 when compared with standard ischemia group; ▲ indicates *P* value is less than 0.05 when compared with diabetic ischemia group.

**Table 4:** Expression level of TNF-*α* (ng/L).

Group	TNF-*α* expression level (ng/L)	
	24 hours after reperfusion	7 days after reperfusion
Sham-operated group	32.23 ± 5.27	25.54 ± 10.20
Standard ischemia group	86.78 ± 21.03^△^	54.21 ± 15.24^△^
Diabetic ischemia group	153.45 ± 30.09^△*∗*^	123.67 ± 24.77^△*∗*^
Diabetic ischemia group and treated with 15d-PGJ2	82.55 ± 22.59^△*∗*▲^	110.49 ± 17.62^△*∗*▲^

Note: △ indicates *P* value is less than 0.05 when compared with sham-operated group; *∗* indicates *P* value is less than 0.05 when compared with standard ischemia group; ▲ indicates *P* value is less than 0.05 when compared with diabetic ischemia group.

**Table 5:** Expression level of IL-1*β* (ng/L).

Group	IL-1*β* expression level (ng/L)	
	24 hours after reperfusion	7 days after reperfusion
Sham-operated group	10.12 ± 1.76	12.34 ± 2.03
Standard ischemia group	42.67 ± 10.72^△^	23.73 ± 9.59^△^
Diabetic ischemia group	80.44 ± 17.28^△*∗*^	55.48 ± 19.33^△*∗*^
Diabetic ischemia group and treated with 15d-PGJ2	37.63 ± 11.52^△*∗*▲^	19.83 ± 7.26^△*∗*▲^

Note: △ indicates *P* value is less than 0.05 when compared with sham-operated group; *∗* indicates *P* value is less than 0.05 when compared with standard ischemia group; ▲ indicates *P* value is less than 0.05 when compared with diabetic ischemia group.

**Table 6:** Percentage of apoptotic cells.

Group	Percentage of apoptotic cells (%)	
	24 hours after reperfusion	7 days after reperfusion
Sham-operated group	25 ± 8.27	28 ± 6.50
Standard ischemia group	65 ± 15.06^△^	58 ± 20.69^△^
Diabetic ischemia group	85 ± 23.34^△*∗*^	73 ± 16.69^△*∗*^
Diabetic ischemia group and treated with 15d-PGJ2	54 ± 17.52^△*∗*▲^	47 ± 12.45^△*∗*▲^

Note: △ indicates *P* value is less than 0.05 when compared with sham-operated group; *∗* indicates *P* value is less than 0.05 when compared with standard ischemia group; ▲ indicates *P* value is less than 0.05 when compared with diabetic ischemia group.

**Table 7:** Quantification of infarcted volume of brain lesion and cerebral edema volume.

Group	24 hours after reperfusion		7 days after reperfusion	
	Infarcted volume (mm^3^)	Cerebral edema volume (mm^3^)	Infarcted volume (mm^3^)	Cerebral edema volume (mm^3^)
Sham-operated group	9.89 ± 1.96	8 ± 2.45	9.53 ± 2.73	7.2 ± 1.42
Standard ischemia group	445.19 ± 78.54^△^	34 ± 9.23^△^	392.19 ± 53.31^△*∗*^	26 ± 8.56^△^
Diabetic ischemia group	601.73 ± 120.25^△*∗*^	46 ± 15.52^△*∗*^	545.73 ± 101.76^△*∗*^	38 ± 12.34^△*∗*^
Diabetic ischemia group and treated with 15d-PGJ2	386.38 ± 98.22^△*∗*▲^	32 ± 8.50^△*∗*▲^	256.27 ± 67.74^△*∗*▲^	19 ± 6.67^△*∗*▲^

Note: △ indicates *P* value is less than 0.05 when compared with sham-operated group; *∗* indicates *P* value is less than 0.05 when compared with standard ischemia group; ▲ indicates *P* value is less than 0.05 when compared with diabetic ischemia group.
